# Report of a Father With Congenital Bilateral Absence of the Vas Deferens Fathering a Child With Beare–Stevenson Syndrome

**DOI:** 10.3389/fgene.2020.00104

**Published:** 2020-02-25

**Authors:** Leonardo C. Ferreira, José H. Dantas Junior

**Affiliations:** ^1^ Department of Biochemistry, Federal University of Rio Grande do Norte, Natal, Brazil; ^2^ Institute of Tropical Medicine of Rio Grande do Norte, Federal University of Rio Grande do Norte, Natal, Brazil; ^3^ University Hospital Onofre Lopes, Urologic Unit, Federal University of Rio Grande do Norte, Natal, Brazil

**Keywords:** Beare–Stevenson, craniosynostosis, paternal age, CBAVD, FGFR2

## Abstract

**Background:**

Apert, Pfeiffer, and Crouzon syndromes are autosomal dominant diseases characterized by craniosynostosis. They are paternal age effect disorders. The association between paternal age and Beare–Stevenson syndrome (BSS), a very rare and severe craniosynostosis, is uncertain. Gain-of-function mutations in *FGFR2* become progressively enriched in testes as men age and were shown to cause these syndromes.

**Case report:**

Here, we describe a child affected with BSS, whose father was 36 years old and had congenital bilateral absence of the vas deferens (CBAVD). The child was heterozygous for the pathogenic *FGFR2* variant c.1124A > G p.Tyr375Cys. By reviewing the literature, we found that BSS fathers are older than BSS mothers (mean age in years: 39 ± 10 vs 30 ± 6, p = 0.006). Male age greater than 34 years and CBAVD are both factors associated with poor spermogram parameters, which may represent an additional selective pressure to sperm carrying *FGFR2* gain-of-function mutations.

**Conclusion:**

These findings are consistent with the hypothesis that BSS is a paternal-origin genetic disorder. Further experimental studies would be needed to confirm this hypothesis.

## Background

Beare–Stevenson Syndrome (OMIM 123790) is a type of craniosynostosis characterized by premature fusion of the cranial sutures associated with *cutis gyrata* (corrugation of skin) as pathognomonic signs. In addition to skin and bone abnormalities, ocular proptosis, choanal atresia, and prominent umbilical stump are frequently found in children affected by this syndrome (Beare et al., 1969; Stevenson et al., 1978). Among the 21 BSS cases reviewed by Wenger et al., 11 died within the first year of life, and at least half of the deaths were due to cardiorespiratory arrest or unexpected sudden death (Wenger et al., 2015).

Mutations in genes encoding fibroblast growth factor receptors (*FGFR*) were shown to cause several craniosynostosis syndromes: Crouzon (*FGFR2*) and Pfeiffer (*FGFR1* and *FGFR2*), Apert (*FGFR2*), Muenke (*FGFR3*), and Jackson–Weiss (*FGFR2*). So far, the genetic causes of BSS are known to be *FGFR2* p.Ser372Tyr and p.Tyr375Cys mutations (Ron et al., 2016), except for a unique BSS case associated with a 63 bp deletion in *FGFR2* exon 8 (Slavotinek et al., 2009). These are autosomal dominant syndromes caused by *de novo* mutations in germ cells in an unaffected parent. It has been estimated that about 80% of *de novo* germline point mutations arise in the male gametes, and a positive correlation exists between mutation rate and paternal age (Acuna-Hidalgo et al., 2016). There are apparently two mechanisms contributing to the paternal age effect (PAE): 1) Increase in DNA copy-error rate as paternal age increases, and 2) Positive selection during spermatogenesis conferred by gain-of-function mutations (Veltman and Brunner, 2012). Nine autosomal-dominant disorders, including Crouzon, Pfeiffer, Apert, and Muenke, have been experimentally shown to be caused by PAE mutations in *FGFR2, FGFR3, HRAS*, *PTPN11*, and *RET*. All five genes act in the RTK-RAS signaling pathway, which is involved with the clonal expansion of spermatogonial stem cells (Goriely et al., 2003; Goriely and Wilkie, 2012).

Paternal age greater than 50 years is associated with 6-, 8-, and 9.5-fold increases in relative risk for Pfeiffer, Crouzon, and Apert syndromes, respectively (Herati et al., 2017). In 1992, Hall et al. described a sixth BSS case and noted a mean paternal age of 36 years (compared to 28 years for the mothers) (Hall et al., 1992). McGaughran et al. reported a BSS case from a 62 year old father, which raised the hypothesis of advanced paternal age as a causal factor of the syndrome (McGaughran et al., 2006). Despite strong biological plausibility, BSS has not been systematically described in the literature as a genetic disease of paternal origin.

Structural abnormalities in the male reproductive tract are a common cause of reduced fertility (Singh et al., 2012). Congenital absence of the vas deferens, which can be unilateral (CUAVD) or bilateral (CBAVD), accounts for 1 to 2% of all male infertility (de Kretser, 1997). Almost 80% of CBAVD cases are associated with a detectable *CFTR* mutation. Among *CFTR* positive individuals, 46% have two mutations, and only 28% have a single mutation (Yu et al., 2012). Besides the CBAVD obstructive azoospermia effect, there is evidence for impaired spermatogenesis in individuals with CBAVD (Meng et al., 2001; Llabador et al., 2015).

This is the first report of a person with Beare–Stevenson Syndrome whose father had CBAVD. Here, we argue that sperm carrying gain-of-function variants in *FGFR2* may be under stronger positive selection during spermatogenesis due to the combined effect of CBAVD and advanced paternal age.

## Case Presentation

### Parents’ Characteristics

The non-consanguineous parents were both 37 years old at the time of the child’s birth (July, 2017). Their family histories showed no genetic diseases, and they were Brazilians. After 12 months of unsuccessfully attempting to conceive, the couple underwent medical evaluation for infertility in 2015. The mother was diagnosed with thrombophilia at that time. Serology tests for HIV, HBV, HCV, syphilis, toxoplasma, rubella IgM, and cytomegalovirus IgM were negative. Rubella and cytomegalovirus IgG were positive.

The father had colorblindness of unknown cause and a history of gastroplasty at age 31 followed by weight loss of approximately 40 kg. Physical examination revealed bilateral absence of vas deferens and absence of the left epididymis. The laboratory analysis detected azoospermia with ejaculated volumes between 1.5 and 2.0 ml; the results confirmed by spermograms performed in different laboratories. Sperm were not identified in any exam even after centrifugation. Scrotal ultrasonography detected absence of the right epididymis and absence of the left epididymis head and body. However, the testes were normal, and there was no evidence of varicocele. Cytogenetics and molecular investigation were performed to identify the cause for the male anatomical defect. G-band karyotyping showed a normal 46, XY chromosomal pattern. Since genetic variants in *CFTR* are the main cause of CBAVD, both mother and father were screened for *CFTR* pathogenic variants to estimate the risk of cystic fibrosis in offspring. Sanger sequencing of the coding region (including exon-intron boundaries) was performed, and deletions/duplications were assessed using multiplex ligation-dependent probe amplification (MRC-Holland SALSA MLPA probemix P091-D1). The parents were negative for pathogenic variants, although intronic variants, including the 5T allele in intron 8, were not investigated. Therefore, *CFTR* could not be ruled out as the cause of CBAVD.

### Assisted Reproductive Technology

The couple underwent two cycles of *in vitro* fertilization (IVF) *via* the intra-cytoplasmic sperm injection (ICSI) method. In the first cycle (December, 2015), sperm were obtained *via* percutaneous epididymal sperm aspiration (PESA) under general anesthesia. The right epididymis was palpable during the procedure, which was inconsistent with the ultrasound result. The PESA was successful on the first puncture, yielding a high number of mobile sperm that were sufficient to proceed to fertilization. The mother had 14 mature eggs that were fertilized, producing four embryos that were frozen on d3 stage to avoid the risk of ovarian hyperstimulation syndrome. The embryos were transferred five months after IVF-ICSI in May (two embryos) and June (the other two embryos), but pregnancy did not occur. The second cycle was started in July 2016. Likewise, sperm were collected from the right epididymis under general anesthesia *via* PESA using two punctures, yielding a large amount of mobile sperm that were used for fertilization. At that time, eight ova were obtained from both ovaries. Six mature ova were fertilized, and three fresh embryos were transferred after three days at the d3 stage. The pregnancy test was positive after 15 days.

### Clinical Findings

Here, we describe a male newborn child with molecular confirmed diagnosis of Beare–Stevenson Syndrome. Several anomalies were detected during prenatal care screening even before birth. Ultrasound examination detected absence of nasal bone at 12 weeks of gestation and brachycephaly at 24 weeks. Both findings were further confirmed by fetal magnetic resonance images. Ultrasound at 27 weeks detected exophthalmia, flat forehead, small femurs, and discrete dilation of the kidneys and cerebral ventricles. A non-invasive prenatal maternal blood test was performed, which was negative for aneuploidies in chromosomes 13,18, 21, X, and Y.

**Figure 1:**
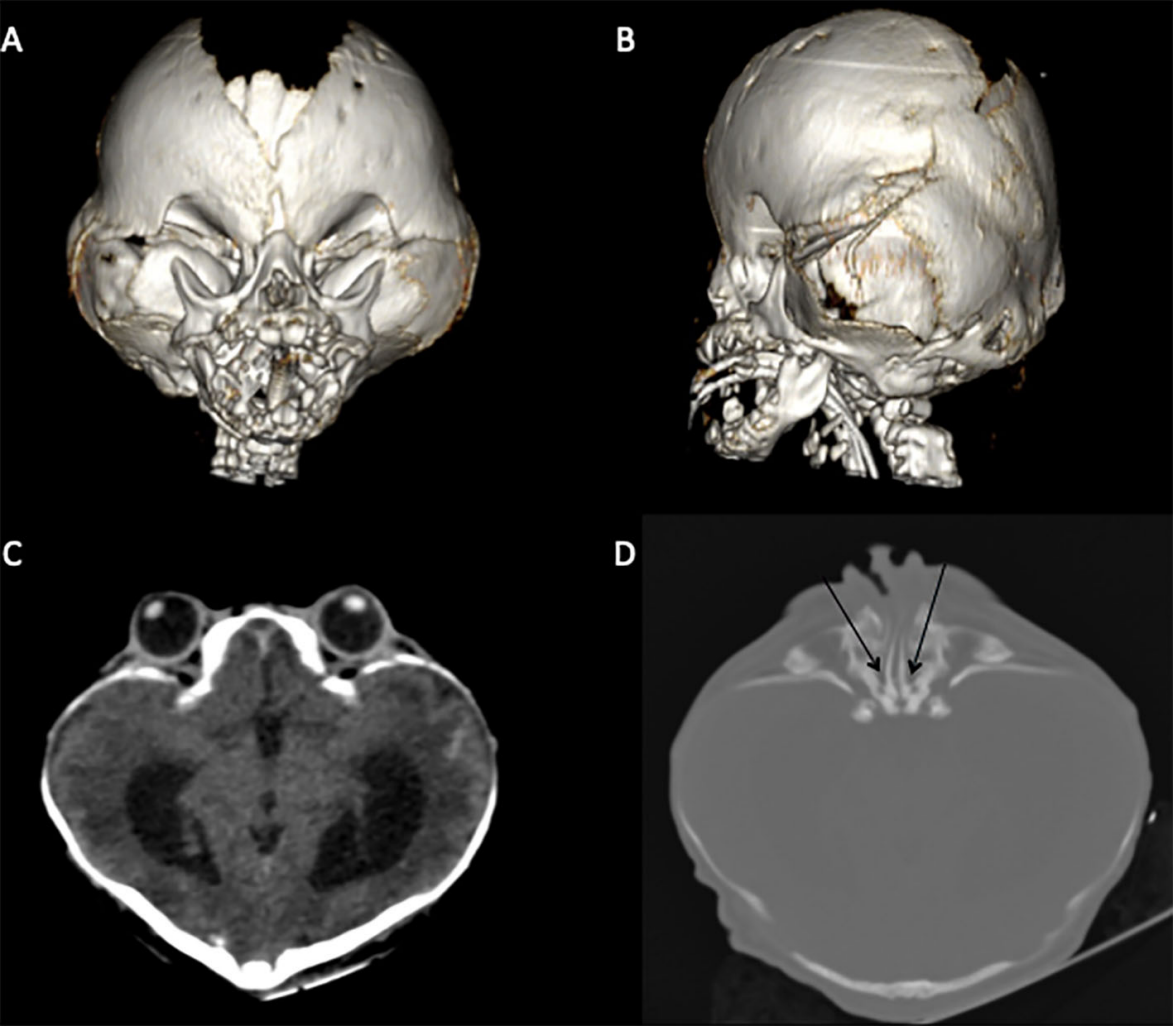
CT scan highlighting classical BSS morphological characteristics. **(A**, **B)** brachycephaly due to premature fusion of coronal and lambdoid sutures, orbital deformity, and midface hypoplasia. **(C)** Hyperteleorbitism and clover-leaf cranial shape. **(D)** Choanal atresia (black arrows).

The mother delivered a male newborn weighing 2,775 g and measuring 47 cm of height at 34.7 weeks gestation *via* Cesarean section. The child presented classical BSS clinical findings: clover-leaf cranial shape, midface hypoplasia, ocular hypertelorism and proptosis, choanal atresia, prominent umbilical stump, hypospadias, short toes, cutis gyrata, and natal tooth. Some of the craniofacial alterations were visualized using computed tomography (CT) at 5 days of life (**Figure 1**).

The newborn was intubated shortly after birth for apnea, and a tracheostomy was performed. He underwent a ventriculo-peritoneal shunt and blepharorraphy to relieve the hydrocephalus and ocular proptosis, respectively. In the 49th day he had a cardiorespiratory arrest due to obstruction of the tracheostomy. He survived, but he had several subsequent seizures. At 60 days of age he had another cardiorespiratory arrest and died.

At 21 days of life, a buccal swab DNA sample was tested for a panel of craniosynostosis-related genes, including *FGFR3*, *FGFR2*, *FGFR1*, and *TWIST1*. Molecular analysis by next generation sequencing (Nextera Exome Capture, Illumina HiSeq) confirmed that the infant was heterozygous for the pathogenic *FGFR2* variant NM_000141.4: c.1124A > G p.Tyr375Cys (rs121913478).

## Discussion

We present the first report of a BSS child whose father has CBAVD. This unique and unlikely finding, in addition to what is known about craniosynostosis genetics and *FGFR2*’s role in spermatogenesis, could be consistent with BSS as a paternal-origin genetic disorder. The main limitations of this report are that the parental origin of the *FGFR2* mutation could not be molecularly determined, and its level in the obtained semen samples was not quantified. To the best of our knowledge, this is the 30th BSS case reported worldwide, and the fourth from Brazil.

We concluded that BSS fathers are significantly older than BSS mothers (mean age 39.1 vs 30.4, p = 0.006) by analyzing the parental ages of BSS cases available in the literature (**Table 1**). However, it is important to acknowledge that differences between paternal vs maternal ages are common across populations and may be biased by the parent’s year of birth. Interestingly, the magnitude of difference between paternal and maternal mean ages is about 5 years for Apert syndrome, with almost 50% of fathers being older than 35 years (Tolarova et al., 1997). It is also noteworthy from **Table 1** that paternal age is more frequently omitted (46.7%) than maternal age (20%), which is consistent with the historical bias of placing greater importance on maternal age when considering rare genetic diseases. We hypothesize a paternal age effect for BSS since Pfeiffer, Crouzon, Apert, and Beare–Stevenson syndromes are all craniosynostoses caused by mutations in *FGFR* genes.

**Table 1:** Parental ages in years (y) from published BSS cases.

Case	Reference	Country	Paternal age (y)	Maternal age (y)
1	(Beare et al., 1969)	North Ireland	40	38
2	(Stevenson et al., 1978)	US	NA	28
3	(Hall et al., 1992)	US	43	33
4	(Hall et al., 1992)	US^1^	30	28
5	(Hall et al., 1992)	US^1^	NA	21
6	(Hall et al., 1992)	US^1^	32	22
7	(Andrews et al., 1993)	Brazil	NA	23
8	(Bratanic et al., 1994)	Slovenia	43	39
9	(Ito et al., 1996)	Japan	NA	25
10	(Przylepa et al., 1996)	US^1^	NA	NA
11	(Krepelová et al., 1998)	Czech	24	24
12	(Wang et al., 2002)	Taiwan	34	31
13	(Akai et al., 2002)	Japan	NA	NA
14	(Izakovic et al., 2003)	US	NA	34
15	(Vargas et al., 2003)	Chile	36	28
16	(Vargas et al., 2003)	Brazil	30	32
17	(Upmeyer et al., 2005)	US	NA	NA
18	(Erol and Eser, 2006)	Turkey	NA	NA
19	(McGaughran et al., 2006)	Australia	62	28
20	(Eun et al., 2007)	Korea	50	32
21	(Fonseca et al., 2008)	Brazil	30	23
22	(Slavotinek et al., 2009)	US	48	40
23	(Tao et al., 2010)	US	48	39
24	(Barge-Schaapveld et al., 2011)	Netherlands	NA	NA
25	(Barge-Schaapveld et al., 2011)	Netherlands	NA	NA
26	(Stater et al., 2015)	US	NA	NA
27	(Wenger et al., 2015)	US	NA	36
28	(Wenger et al., 2015)	US	NA	22
29	(Ron et al., 2016)	US^1^	NA	36
30	(Ferreira et al., 2020)	Brazil	37	37
**Missing age information (%)**			**14/30 (46.7)**	**6/30 (20.0)**
**Mean age (standard deviation)**			**39.1 (10)**	**30.4 (6.3)**

P-value = 0.05 for comparison of missing age information rate Paternal vs Maternal (chi-squared test). P-value = 0.006 for comparison of means Paternal vs Maternal (t-test). NA, parental age not available at the original publication.

1 It refers to corresponding author’s location. It was not mentioned the location where the case was born.

Unlike oogenesis, spermatogenesis is a continuous process occurring from male puberty throughout life. There is an evident negative impact of advanced paternal age on sperm parameters. Based on semen analysis of 4,822 men, Stone et al. verified that daily sperm production significantly declines after 34 years of age (Stone et al., 2013). Hypospermatogenesis was histopathologically detected in 33/54 (61.1%) males with CBAVD that had been subjected to testicular sperm extraction (Llabador et al., 2015). By analyzing 85 CBAVD patients who had undergone PESA-ICSL, Elhanbly et al. demonstrated that paternal age was negatively correlated with sperm count, motility, vitality, and normal sperm morphology. They estimated a reduction in the number of retrieved-sperm of 0.53 million per 10 years (Elhanbly et al., 2015). In addition to age and CBAVD, a variety of metabolic (*e.g.* obesity and diabetes) and environmental (*e.g.* cadmium and dioxins) factors affect spermatogenesis (Neto et al., 2016).

The transcriptional dynamics of spermatogenesis are consistent with a five-stage process (Stage 0–Stage 4) involving cell types from five niches (Leydig, myoid, Sertoli, endothelial, and macrophage) (Guo et al., 2018). In this context, *FGFR3* and *FGFR2* were described as early spermatogonial markers (Guo et al., 2017; Guo et al., 2018). Gain-of-function mutations in *FGFR2* offer a selective advantage for spermatogonial cells by favoring clonal expansion in the testes, yielding enrichment in mutated sperm by at least 100-fold (Goriely et al., 2009). Thus, Goriely et al. coined the term “selfish spermatogonial selection” to describe the increase in the number of mutant sperm over time (*i.e.* male aging) caused by the mutation-driven clonal expansion process (Maher et al., 2018). Of note, Maher et al. (2018) identified a mutational clone in human testes for the *FGFR2* Ser372Cys, one of the mutations known to cause BSS. In situations like advanced paternal age and CBAVD, which are both characterized by poor spermogram parameters, the before-mentioned clonal expansion process would lead to a relative increase in the number of mutated sperm, compared to situations consistent with normal spermatogenesis. Therefore, we hypothesize that spermatogenesis in a 36 years old man with CBAVD is under additional selective pressure, making the *FGFR2* p.Tyr375Cys mutation even more advantageous to sperm.

*FGFR2* is a pleiotropic gene widely expressed throughout the human body. The highest expression is found in the spinal cord, colon, uterus, and skin (data from GTExPortal, https://gtexportal.org/home/gene/FGFR2). Despite its ubiquitous presence in human tissues, *FGFR2* expression is crucial for proper lung-branching morphogenesis (Arman et al., 1999). Normal tracheal homeostasis depends on a suitable FGFR2 signaling pathway to promote asymmetric self-renewing division generating one basal cell and one luminal cell per basal cell division (Balasooriya et al., 2017). Tracheal cartilaginous sleeve (TCS), a rare life-threatening condition, has been found in some individuals affected by mutations in *FGFR* genes (Pickrell et al., 2017; Wenger et al., 2017). Notably, Wenger et al. identified TCS in 100% of *FGFR2* p.Try290Cys mutated children (Wenger et al., 2017), in addition to a previous report of TCS in a BSS case (Wenger et al., 2015). Seventy-five percent of TCS cases require tracheostomy, a procedure that is imperative to minimize the risk of sudden death in BSS-affected individuals. Therefore, medical staff should investigate TCS in BSS patients, and they must be prepared to surgically manage TCS (Stater et al., 2015).

Very recently, Zhang et al. developed a non-invasive prenatal sequencing test based on the unique molecular indexing method to diagnose dominant monogenic disorders. This test successfully identified cases of *FGFR2*-related craniosynostosis, including Apert (*FGFR2*: c.758C > G), Pfeiffer (*FGFR2*: c.870G > T), and Crouzon (*FGFR2*: c.1032G > A) syndromes, and it accurately distinguished those cases from other *FGFR3*-related skeletal disorders (*e.g.* thanatophoric dysplasia and achondroplasia) (Zhang et al., 2019). This diagnostic approach would be useful in situations like the one presented in this case report. The molecular confirmation of BSS at the prenatal stage would allow both medical staff and family to cope with this life-threatening syndrome. Finally, two pharmacological approaches targeting different points of the FGF signaling pathway have been successfully used in *ex vivo* organ culture (Balek et al., 2017) and in BSS mice harboring the human-analog *FGFR2* p.Tyr375Cys mutation (Wang et al., 2012). We envision that prenatal molecular diagnosis may be an essential step toward the early application of therapeutic tools, aiming to ameliorate BSS symptoms and to extend life expectancy in these cases.

## Data Availability Statement

The DNA sequencing service was ordered by the study participants and performed by Mendelics, a private company. Therefore, we had no access to raw data.

## Ethics Statement

This study involves three participants (i.e. father, mother and child). The parents provided written informed consent allowing their child to be included in this case report publication. In addition, as the parents are participants themselves, both the father and mother gave written informed consent to be included as study participants. The protocol was reviewed and approved by the Ethical Committee from Federal University of Rio Grande do Norte (ethical approval: 3.613.457).

## Author Contributions

LF conceptualized the case report into a scientific question, reviewed the literature, and drafted the manuscript. JD conducted clinical and surgical procedures in the male patient (i.e. father), critically reviewed the manuscript, and added important intellectual contributions.

## Funding

This study was financed in part by the Coordenação de Aperfeiçoamento de Pessoal de Nível Superior - Brasil (CAPES) - Finance Code 001.

## Conflict of Interest

The authors declare that the research was conducted in the absence of any commercial or financial relationships that could be construed as a potential conflict of interest.
